# Psychometric properties and factorial invariance of the Farsi version of the Stress Mindset Measure

**DOI:** 10.3389/fpsyg.2022.945673

**Published:** 2022-09-09

**Authors:** Yaser Tedadi, Yalda Daryani, Hossein Karsazi

**Affiliations:** Faculty of Psychology and Educational Sciences, University of Tehran, Tehran, Iran

**Keywords:** stress mindset, Stress Mindset Measure, psychometric properties, factorial invariance, validity, reliability

## Abstract

The Stress Mindset Measure consists of eight items to assess whether individuals hold a stress-is-enhancing or a stress-is-debilitating mindset. The current research is a cross-sectional study and aimed to investigate the factor structure, internal consistency reliability, and construct and convergent validity of the Farsi version of the Stress Mindset Measure (SMM). Prior to conducting the study, forward and backward translations of the SMM were performed. Using the convenience sampling method, we recruited 400 none-clinical sample (161 men and 239 women; aged 18 to 69). We utilized SPSS version 24, Amos, and Mplus 7.1 software to analyze the data. Results revealed satisfactory reliability and validity indexes for the Farsi version of the Stress Mindset Measure. The internal consistency of the Farsi version of the Stress Mindset Measure was in the excellent range (α = 0.87). The results of the confirmatory factorial analysis revealed two factors of the Stress Mindset Measure instead of the single factor suggested by the previous studies (fitness indices for the two-factor model were RMSEA = 0.78, CFI = 0.96, and TLI = 0.94). Moreover, we found that the stress-is-debilitating mindset is positively associated with stress (*r* = 0.233), depression (*r* = 0.163), and anxiety (*r* = 0.197). However, this mindset has been found to have no significant relationship with cognitive strategies of emotion regulation and life satisfaction. Also, findings showed no significant correlation between the stress-is-enhancing mindsets and the other variables. The results of this study suggest that the Farsi SMM has proper psychometric properties to assess stress mindsets.

## Introduction

Stress is defined as an experienced tension when individuals perceive that the demands from external events are beyond their coping capacity (Lazarus and Folkman, 1984; Lovallo, 2015). Numerous studies portraying the detrimental effects of stress have consolidated negative cultural narratives around stress, proposing that stress must be reduced or removed (Crum et al., 2020). Over the years, stress has been cited as causing cardiovascular diseases (Juster et al., 2010), brain aging (Jefferson et al., 2010), and cognitive impairments (Schwabe and Wolf, 2010). However, the new line of research in the science of stress has shed light on the positive outcomes of stress by introducing the concept of stress mindsets (Keller et al., 2012; Crum et al., 2013; Nabi et al., 2013). Mindsets are lenses that filter and categorize the information people receive every day. Mindsets determine how individuals experience, understand and respond to the surrounding stimuli (Dweck, 2013). Stress mindset, a recently introduced concept, refers to the belief about whether stress is enhancing or debilitating for cognitive, emotional and performance outcomes. This concept shifts our attention to the fundamental role of the belief and attitude toward the effects of stress on various aspects of our wellbeing. Correlational studies and randomized controlled trials on stress mindsets have demonstrated that the stress-is-enhancing mindset-believing that stress increases health, vitality, learning, growth, and performance- is linked to reduced symptoms of anxiety and depression, improved self-reported health and energy levels, greater life satisfaction, and performance at work (Crum et al., 2013, 2017). On the other side of the continuum, there is the stress-is-debilitating mindset, which holds the belief that stress negatively affects performance, health, and wellbeing. Stress-is-debilitating mindset is more prevalent in individuals (Clark, 2003; Kinman and Jones, 2005) since the mass media constantly underpins the negative ramifications of stress (Cohen et al., 2007). Specifically, research studies have revealed that the extent to which people believe that stress is debilitating is positively correlated with the rate of mortality and morbidity (Keller et al., 2012; Nabi et al., 2013). Conversely, further studies have displayed constructive consequences of stress (Podsakoff et al., 2007). For instance, it has been shown that introducing a stress-is-enhancing mindset improves physiological functioning (Jamieson et al., 2010, 2013), as well as escalating work performance and self-reported health (Crum et al., 2013).

One of the implications of this recent notion toward stress is that individuals can be placed on the spectrum of stress mindset with the stress-is-enhancing mindset on one side and the stress-is-debilitating on the other side. Further research studies have manipulated the stress mindset with the mission of increasing the extent to which people adhere to a stress-is-enhancing mindset (Crum et al., 2013, 2017). In their pioneering study, Crum et al. (2017) found that activating a stress-is-enhancing mindset increases dehydroepiandrosterone sulfate secretion, cognitive flexibility, positive affect, and attention toward positive stimuli. One of the crucial implications of this study is that the stress mindset can be manipulated through straightforward interventions. These interventions may be applicable to non-clinical issues such as managing stress during a pandemic (Hagger et al., 2020). Also, Crum et al. (2013) have elucidated that stress is not always enhancing, but it can be utilized to be enhancing. This notion is in line with the theory of mindsets, describing mindsets as individuals’ beliefs about fundamental attributes such as intelligence and personality- whether the person considers them to be fixed or malleable (Dweck et al., 1995; Dweck and Yeager, 2019). Studies have revealed that when people change their mindsets from fixed to growth, they experience improvements in their functions and achievements (Yeager et al., 2019).

Whether we want to manipulate the stress mindset or conduct a correlational study, we first need to determine the individuals’ position on the stress mindset spectrum. To this end, we need to use the Stress Mindset Measure (SMM). This measure is used to determine the subjective meaning people ascribe to stress and decide how stress influences their performance, health, and wellbeing (Crum et al., 2013). The SMM consists of eight items and asks the participants to show their agreement or disagreement with sentences about the effects of stress on learning and growth, vitality, performance, health, and productivity (Crum et al., 2013). Extensive studies have validated and utilized the SMM in various populations, such as college students (Crum et al., 2017; Goyer et al., 2018), firm employees (Crum et al., 2013, 2018), and Navy SEALs (Smith et al., 2020). Crum et al. (2013) created two versions of the measure in the validation study of SMM. The first version consisted of beliefs about the general nature of stress (SMM-G), while the second one consisted of stress when a specific stressor was present (SMM-S). These two versions were shown to be internally consistent (Cronbach’s alpha for the SMM-G was 0.86 and for the SMM-S was 0.80) and the confirmatory factor analyses confirmed the single structure of SMM.

Studies have shown that mindsets can lead us to a better understanding of the effects of stress on physical and mental health (Crum et al., 2017). Therefore, validating the measures that assess the stress mindsets is of critical importance. Although previous studies support the psychometric properties of the SMM in Western countries, the generalization of these results to countries with different cultures, such as Iran, can be problematic. This concern is salient in studying stress, given that the process that triggers and perpetuates stress must be evaluated from a cultural perspective (Han et al., 2022). Also, stress is experienced, described, and labeled in various ways in different cultures (Moheb and Ram, 2010; Shokri et al., 2012; Lee et al., 2021). Thus, it is vital to investigate the psychometric properties of the SMM in Iran. We designed this study to investigate the psychometric properties and factorial invariance of the SMM in Iranian culture.

In the present study, we aimed to provide a fluent Farsi translation of SMM and study the psychometric properties of the measure in the Iranian population. To adapt the SMM to the Iranian context and study the reliability and validity of the measure for assessing the stress mindsets, we translated the measure into Farsi, hired 400 none-clinical participants and analyzed the data for inter-item correlations, confirmatory factor analysis, internal consistency reliability, and factorial invariance.

## Materials and methods

### Procedure

The current research was a cross-sectional study. To determine the sample size, we based our work on research studies suggesting having at least a 10:1 ratio of participants to an item for factor analysis (Everitt, 1975; Costello and Osborne, 2005). Given that the SMM has 8 items, the minimum sample size for our study was estimated to be 80. Considering that the overall number of participants was 400, it can be concluded that the ultimate sample size was appropriate.

We recruited participants via advertisements of social networks and provided information about the study. Data was gathered online, using Porsline, an Iranian platform for creating online questionnaires and conducting studies in the field of social sciences.

### Participants

Participants responded to the SMM questions after giving consent and answering some demographic questions. The inclusion criteria were: (a) at least 18 years of age and older, (b) being able to speak and read in Farsi, and (c) no history of severe mental and physical disorders. The exclusion criteria were: (a) answering the questions incompletely.

### Translation

All SMM items were translated into Farsi using the standard back translation technique (Brislin, 1970). Specifically, the first and the second authors translated the SMM into Farsi from the original English version. In the next step, two independent translators translated the SMM back into English. Before using the translated measure in the research procedure, we sent the translation files to the mind and body lab at Stanford University, directed by Alia Crum, the creator of the SMM. After being confirmed and published on the mind and body lab website, we used the Farsi translation in the study.

### Measures

In the present research, we used three other measures to investigate the criterion and concurrent validity of the Farsi version of the SMM. For this purpose, we used the following measures:

1. Stress Mindset Measure (SMM; Crum et al., 2013). In this measure, eight items are presented to the participants and they are asked to show their agreement or disagreement to them on a five-point Likert scale (from 0 = strongly disagree to 4 = strongly agree). Four items assess the stress-is-enhancing mindsets (e.g., Experiencing stress facilitates my learning and growth) and the four others assess the stress-is-debilitating mindset (e.g., The effects of stress are negative and should be avoided). Cronbach’s alpha for this measure is 0.87.
2. Satisfaction With Life Scale (SWLS; Diener et al., 1985; Farsi version: Bayani et al., 2007). This scale uses five items (e.g., In most ways, my life is close to my ideals) and a seven-point Likert scale (from 1 = strongly disagree to 7 = strongly agree) to assess the reported level of satisfaction in life. The reported Cronbach’s alpha for the Farsi version is 0.83. In this study, we found Cronbach’s alpha of 0.826 for the scale.
3. Depression Anxiety Stress Scales-21 (DASS-21; Lovibond and Lovibond, 1995; Farsi version: Samani and Joukar, 2007). In this scale, the participants report the presence of depression, anxiety, and stress symptoms in the last week (e.g., I was aware of dryness in my mouth) on a Likert scale (from 0 = did not apply to me at all to 3 = applied to me very much or most of the times). In the study conducted by Samani and Joukar (2007), high internal consistency levels were found for the scales (α = 0.81, α = 0.74, and α = 0.78 respectively). We found Cronbach’s alpha of 0.845 for stress, 0.813 for anxiety, and 0.837, for depression.
4. Emotion Regulation Questionnaire (ERQ; Gross and John, 2003; Farsi version: Qasempour et al., 2012). This 10-item self-report questionnaire assesses the use of cognitive reappraisal and expressive suppression as common strategies to alter emotions. Participants respond to each item (e.g., I control my emotions by not expressing them) using a seven-point Likert scale (1 = strongly disagree to 7 = strongly agree). Our results revealed Cronbach’s alpha of 0.706 for reappraisal and 0.740 for expressive suppression strategies.

Confirmatory factor analysis was applied to assess the structure of the SMM. We used maximum likelihood estimation for assessing the parameters of the assumed model. We investigated the fitness of the model by the Root Mean Square Error of Approximation (RMSEA), Comparative Fit Index (CFI), Tucker-Lewis Index (TCI), Goodness of Fit Index (GFI), Expected Cross-Validation Index (ECVI), Normed Fit Index (NFI), and Incremental Fit Index (IFI). We measured factorial invariance of the SMM between men and women by multigroup confirmatory factor analysis (Kline, 2015). Also, internal consistency was evaluated by Cronbach’s alpha coefficient and convergent validity with other scales was assessed by Pearson’s correlation.

## Results

This study was conducted by hiring 400 healthy individuals (aged 18 to 69). 239 of the participants were women (59.8%) and 161 were men (40.3%). The mean age of the participants was 31.89 (SD = 10.80).

### Inter-item correlations

First, we examined the inter-item correlations among the eight SMM items. After reversing the negative items, the findings indicated that the inter-item correlations ranged from 0.31 to 0.62 (see Table 1). According to de Vaus (2004), the inter-item correlations between 0.30 and 0.70 were considered acceptable. Results reveal proper item homogeneity and SMM items measure the same concept.

**TABLE 1:** Stress Mindset Measure inter-item correlations.

Item no.	1	2	3	4	5	6	7	8
1	1							
2	0.328**	1						
3	0.436**	0.378**	1					
4	0.317**	0.527**	0.378**	1				
5	0.394**	0.469**	0.469**	0.551**	1			
6	0.360**	0.479**	0.442**	0.532**	0.484**	1		
7	0.460**	0.411**	0.489**	0.610**	0.622**	0.497**	1	
8	0.461**	0.484**	0.357**	0.574**	0.444**	0.533**	0.476**	1

The symbol ** indicates the significance of correction.

### Confirmatory factor analysis

**FIGURE 1:**
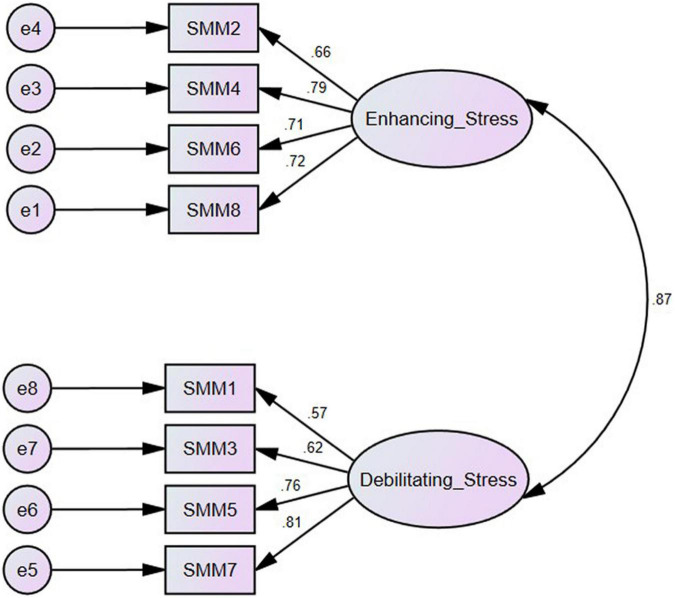
The two-factor solution model for the Farsi version of the SMM.

Confirmatory factor analysis was conducted by Amos version 24 to examine the construct validity of the measure. According to the recommended model designed by Crum et al. (2013), in which all the eight items loaded on a single stress mindset factor, a first-order single-factor model was created. Next, we examined a first-order two-factor model based on the two elements of the SMM (see Figure 1). Four items (q2, q4, q6, and q8) loaded on a factor that evaluated stress-is-enhancing mindset. The other four items (q1, q3, q5, and q7) loaded on a factor that measures the stress-is-debilitating mindset. We used various indexes to assess the fitness of the models. First, we analyzed the Chi-square ratio. The Chi-square ratios lower than three indicate the goodness of fit of the model (Kline, 2010). Also, we examined other indexes such as the expected cross-validation index (ECVI; Schreiber et al., 2006); Tucker-Lewis index (TLI; Tucker and Lewis, 1973), the goodness of fit index (GFI; Joreskog and Sorbom, 1984), the comparative fit index (CFI; Bentler, 1990), and the root means square error of approximation. Based on previous studies, RMSEA values below 0.08 indicate a good model fit (Hu and Bentler, 1999). Further, CFI, GFI, and TLI values greater than 0.90 showed acceptable model fit, whereas values higher than 0.95 display great model fit. It should be noted that ECVI is mainly used to compare various models and the smaller values indicate better model fit.

Our findings revealed that the Chi-square ratio was higher than three for both models due to the big sample size. For the single-factor model, CFI, GFI, TLI, NFI, and IFI values were greater than 0.90, which indicates a good model fit. Also, CFI, GFI, and IFI values for the two-factor model were 0.96, indicating a great model fit. Given that for the two-factor model, CFI, TLI, GFI, and NFI values were higher than 0.90, and RMSEA value was less than 0.08, and ECVI values were smaller than the first-factor model. It can be concluded that the fit indices for the two-factor model are better than the single-factor model (see Table 2).

**TABLE 2:** Fit indices in confirmatory factor analysis for a single-factor and a two-factor.

Model	χ^2^	*p*	df	χ^2^/df	CFI	TLI	GFI	NFI	IFI	RMSEA [90% CI]	ECVI
Single-factor	100.70	0.000	20	5.03	0.937	0.911	0.941	0.923	0.937	0.101 [0.082 −0.121]	0.334
Two-factor	65.22	0.000	19	3.43	0.964	0.947	0.961	0.950	0.964	0.078 [0.058 −0.099]	0.250

### Internal consistency reliability

We calculated the Cronbach’s alpha coefficient to evaluate the internal consistency reliability of the two factors and the total score of the SMM. The total alpha score for the SMM was 0.87, which indicates high internal consistency reliability. Also, the alpha coefficient for the stress-is-enhancing mindset was 0.81 and for the stress-is-debilitating mindset was 0.78 indicating great internal consistency.

### Factorial invariance

To investigate the factorial invariance of the SMM, we employed multi-group confirmatory factor analysis between men and women. We used three models of invariance for the analysis. The configural model considers the structure and pattern of the factors constant. In the metric model, factor loadings between groups are considered equivalent as well as the structures. As it is apparent in Table 3, Chi-square is not significant when comparing the metric model with the configural model. Also, the ΔCFI value is lower than the cut point, which is 0.01, indicating metric factorial invariance between men and women. In the scalar model, the intercepts between two groups are considered equivalent, as well as the structure and factor loadings. Chi-square not being significant and the small values of ΔCFI in the scalar model compared to the metric model shows the scalar factorial invariance in men and women (see Table 3).

**TABLE 3:** Factorial invariance across gender for the SMM model.

Model	χ^2^	df	CFI	Model comparison	Δχ^2^	Δdf	Sig	Δ CFI
1. Configural	86.82	38	0.960	–	–	–	–	–
2. Metric	94.20	44	0.958	2 vs. 1	7.38	6	*p* = ns	0.002
3. Scalar	100.12	50	0.959	3 vs. 2	5.92	6	*p* = ns	0.001

### Convergent validity

In the final step, we tested the correlation of the SMM items with the pertinent positive and negative concepts (see Table 4). Results show that the stress-is-debilitating mindset is positively associated with stress (*r* = 0.233), depression (*r* = 0.163), and anxiety (*r* = 0.197). However, this mindset has been found to have no significant relationship with cognitive strategies of emotion regulation and life satisfaction. Also, findings displayed no significant correlation between the stress-is-enhancing mindsets and the other variables.

**TABLE 4:** Bivariate correlations between DAS, ERQ, and SWLS with the SMM subscales.

Model	Enhancing stress	Debilitating stress
Depression	−0.053	0.163**
Anxiety	−0.040	0.197**
Stress	−0.085	0.233**
Cognitive reappraisal	0.044	0.053
Suppression	−0.053	0.094
Satisfaction With Life	0.075	−0.068

The symbol ** indicates the significance of correction.

## Discussion

The current research aimed to examine the psychometric properties and factorial invariance of the Farsi version of the SMM. The results depicted that the Farsi translation of the SMM conveys the concepts that the creators of the measure intended. However, our findings showed a different structure of the SMM in Iran. Contrary to the single stress mindset factor that Crum et al. (2013) proposed, we identified two factors. These factors indicated individuals’ beliefs about the effects of stress, whether stress-is-enhancing or stress-is-debilitating. In other words, we found that these two mindsets are independent rather than the constituents of a universal stress mindset. One explanation for this could be the reversed scoring of the stress-is-debilitating items. Studies have revealed that utilizing positive items as well as reversed items creates secondary sources of variance and this hinders the unidimensionality of the scale (Suárez Álvarez et al., 2018). In this vein, the results of a study investigating the psychometric properties of the SMM in a Greek sample have also identified two factors instead of a single stress mindset factor (Karampas et al., 2020).

The first factor, “Enhancing Stress,” includes items that capture the belief that stress has enhanced consequences for various stress-related outcomes such as performance and productivity, health and wellbeing, and learning and growth. It includes four items with significant factor loadings and high internal consistency. The second factor, “Debilitating Stress,” reflects the belief that stress has debilitating consequences for those outcomes. All four items had significant factor loadings on their underlying constructs and satisfactory internal consistency. The results showed a high correlation between Enhancing and Debilitating Stress, with coefficients of 0.87. So our results revealed that stress mindset is a variable with two distinct categories that influence the stress response.

Also, we found adequate configural, metric, and scalar invariance of the instrument in the analysis of factorial invariance. These results show that the Farsi Stress Mindset Measure evaluates the stress mindset with the exact structure and meaningfully across men and women and the total sample.

Moreover, findings revealed that the stress-is-debilitating mindset is positively associated with stress, depression, and anxiety, meaning that people who hold a stress-is-enhancing mindset are more likely to suffer from the symptoms of these disorders. More specifically, the stress-is-debilitating mindset had the strongest correlation with the stress symptoms. Stress-is-enhancing mindset, on the other hand, was not associated with the symptoms of mental disorders. These results are in accordance with the prior studies revealing that individuals with a stress-is-debilitating mindset (not a stress-is-enhancing mindset) are at a higher risk of experiencing mental health concerns (Keech et al., 2018; Huebschmann and Sheets, 2020).

This study had a few limitations. First, because the data collection procedure took place in the university context and participants were selected from the university students’ population, the findings will only indicate the stress mindset of a restricted portion of the Iranian population. Therefore, conducting a similar study using a more inclusive sample is recommended. Second, we did not ask questions about the ethnicity and cultural background of the participants. Although all participants were Iranian, their ethnicities were not specified. Given that there are various ethnicities and linguistic backgrounds coexist in Iran, the lack of these data could be considered a limitation. Investigating the psychometric properties of the SMM in other cultures and languages in Iran is recommended for future studies.

Based on our results, we can consider the SMM an appropriate tool to measure stress mindsets. The Farsi version of the SMM could be applied to assess the stress mindset in both research and clinical realms.

## Conclusion

In conclusion, the results of the present study indicate that the Farsi version of the SMM is a valid and reliable tool to measure the stress mindset in the Iranian population. Also, our findings corroborate the notion that the stress mindset can determine the psychological symptoms that individuals experience when they are stressed.

## Data availability statement

The data and materials that support the findings of this study are openly available in figshare at https://doi.org/10.6084/m9.figshare.19362326.

## Ethics statement

The studies involving human participants were reviewed and approved by the University of Tehran, Ethics Committee of the Faculty of Psychology and Educational Sciences. The patients/participants provided their written informed consent to participate in this study.

## Author contributions

YT and YD performed the translation, material preparation, and data collection. HK analyzed the data and collaborated in writing the first draft of the manuscript. All authors contributed to the study conception and design, read, and approved the final manuscript.
